# Functional expression and characterization of cinnamic acid 4-hydroxylase from the hornwort *Anthoceros agrestis* in *Physcomitrella patens*

**DOI:** 10.1007/s00299-020-02517-z

**Published:** 2020-02-13

**Authors:** Julia Wohl, Maike Petersen

**Affiliations:** Institut für Pharmazeutische Biologie und Biotechnologie, Philipps-Universität Marburg, Robert-Koch-Str. 4, 35037 Marburg, Germany

**Keywords:** Bryophytes, Cinnamic acid 4-hydroxylase (C4H) CYP73A260, Cytochrome P450, NADPH:cytochrome P450 reductase (CPR or POR), Heterologous expression, Phenylpropanoid pathway

## Abstract

**Key message:**

Cinnamic acid 4-hydroxylase from the hornwort *Anthoceros agrestis* (AaC4H) was functionally expressed in the moss *Physcomitrella patens* and characterized at biochemical and molecular levels.

**Abstract:**

Cinnamic acid 4-hydroxylase (C4H), a cytochrome P450-dependent hydroxylase, catalyzes the formation of 4-coumaric acid (=4-hydroxycinnamic acid) from *trans*-cinnamic acid. In the hornwort *Anthoceros agrestis* (Aa), this enzyme is supposed to be involved in the biosynthesis of rosmarinic acid (a caffeic acid ester of 3-(3,4-dihydroxyphenyl)lactic acid) and other related compounds. The coding sequence of AaC4H (CYP73A260) was expressed in the moss *Physcomitrella patens* (Pp_AaC4H*)*. Protein extracts from the transformed moss showed considerably increased C4H activity driven by NADPH:cytochrome P450 reductase of the moss. Since *Physcomitrella* has own putative cinnamic acid 4-hydroxylases, enzyme characterization was carried out in parallel with the untransformed *Physcomitrella* wild type (Pp_WT). Apparent *K*_m_-values for cinnamic acid and NADPH were determined to be at 17.3 µM and 88.0 µM for Pp_AaC4H and 25.1 µM and 92.3 µM for Pp_WT, respectively. Expression levels of AaC4H as well as two *Physcomitrella patens* C4H isoforms were analyzed by quantitative real-time PCR. While PpC4H_1 displayed constantly low levels of expression during the whole 21-day culture period, AaC4H and PpC4H_2 increased their expression during the first 6–8 days of the culture period and then decreased again. This work describes the biochemical in vitro characterization of a cytochrome P450-dependent enzyme, namely C4H, heterologously expressed in the haploid model plant *Physcomitrella patens*.

**Electronic supplementary material:**

The online version of this article (10.1007/s00299-020-02517-z) contains supplementary material, which is available to authorized users.

## Introduction

One of the early steps of the phenylpropanoid pathway is catalyzed by cinnamic acid 4-hydroxylase (C4H; EC 1.14.14.91). The enyzme is one of the best characterized cytochrome P450 hydroxylases from higher plants (Werck-Reichhart 1995). C4H has already been biochemically characterized from pea seedlings as early as 1967 (Russell and Conn 1967; Russell 1971). It catalyzes the hydroxylation (with NADPH and O_2_ as cosubstrates) of the aromatic ring of *t*-cinnamic acid in *para*-position leading to 4-coumaric acid (4-hydroxycinnamic acid).

Petersen (2003) characterized C4H from cell cultures of the hornwort *Anthoceros agrestis* Paton (Anthocerotaceae) as one of the first cytochrome P450 enzymes from lower plants. Suspension-cultured cells of *Anthoceros agrestis* can accumulate around 5% of the dry weight as rosmarinic acid (Vogelsang et al. 2006), a caffeic acid ester of 3-(3,4-dihydroxyphenyl)lactic acid. Furthermore, other lignan-like compounds such as anthocerotonic acid and megacerotonic acid can be found in hornworts (Takeda et al. 1990a, b; Trennhäuser 1992). Therefore, hydroxycinnamic acid units must be present for the biosynthesis of these compounds.

Bryophytes as non-vascular land plants are devided into three divisions: liverworts (Marchantiophyta), mosses (Bryophyta) and hornworts (Anthocerotophyta) (Troitsky et al. 2007). They are all characterized by the absence of flowers and the formation of only one spore capsule on the sporophyte (monosporangiate), a distinct alternation of generations with a dominant haploid gametophyte and a diploid unbranched sporophyte and the absence of lignified vascular tissue, although lignin-like epitopes were detected in cell walls (Espiñeira et al. 2011). The phylogenetic relationships between the bryophytes and the vascular plants are still under discussion, placing the hornworts either as sister group to the tracheophytes or the green algae or hornworts together with liverworts and mosses as sister clade to the other land plants (Szövenyi et al. 2015; Puttick et al. 2018).

The moss *Physcomitrella patens* (Hedw.) Bruch & Schimp. (Funariaceae) has been investigated as an appropriate model system for differentiation analysis and the investigation of gene function in molecular and cellular development due to its high and efficient homologous recombination. Since the moss gametophyte is haploid, gene function analysis through targeted knockout results in immediately visible phenotypes (Cove 1992; Cove and Knight 1993; Schaefer and Zrÿd 1997). In addition, mosses are recognized as similar to higher plants in terms of gene content, expression, and regulation (Reski 1999). *Physcomitrella patens* is suitable for cheap and high-volume production of recombinant proteins. It is now also in use in bioreactors for the production of complex biopharmaceutical products (Reski et al. 2015, 2018). As a eukaryotic organism, it has the ability to perform postranslational protein modifications, such as the formation of disulfide bridges and complex glycosylation reactions (Koprivova et al. 2003). Some genes of plant specialized metabolism have already been successfully expressed in *Physcomitrella patens*, e.g., taxadiene synthase from *Taxus brevifolia* (Anterola et al. 2009), sclareol synthase from *Salvia sclarea* (Pan et al. 2015) and patchoulol synthase from *Pogostemon cablin* or santalene synthase from *Santalum album* (Zhan et al. 2014). The full biosynthetic system for artemisinin production, including a cytochrome P450-catalyzed step, has also successfully been transferred to *Physcomitrella patens* (Khairul Ikram et al. 2017).

Our research aims at the molecular and biochemical elucidation of the biosynthetic pathway of rosmarinic acid in hornworts in comparison to higher plants, namely members of the Lamiaceae. This will shed light on the mono- or polyphyletic evolution of the phenylpropanoid pathway and rosmarinic acid biosynthesis in land plants. We here describe the successful amplification of a *C4H* sequence (CYP73A260) from the hornwort *Anthoceros agrestis.* Heterologous expression of *AaC4H* in *Saccharomyces cerevisiae* failed (data not shown), presumably because of the high GC content and/or different codon usage; thus, we aimed to find an alternative expression host. *AaC4H* was transferred to *Physcomitrella patens* by protoplast transformation and integration of the coding sequence into the moss genome by homologous recombination. AaC4H expressed in *Physcomitrella patens* (Pp_AaC4H) was biochemically characterized in comparison to *Physcomitrella*’s own C4H. This is the first report of the in vitro biochemical characterization of C4H, a cytochrome P450, a membrane-anchored protein, heterologously expressed in *Physcomitrella patens*.

## Materials and methods

### Plant cell cultures

Cell suspension cultures of *Anthoceros agrestis* were cultivated as described previously (Petersen 2003).

*Physcomitrella patens* (provided by Dr. Stefan Martens, Fondazione Edmund Mach, Italy) protonemata were cultivated in 50 ml BCD medium (Cove et al. 2009a) in 250-ml Erlenmeyer flasks. For maintenance, the tissue was disrupted with a sterilized tissue blender (Omni International) for 30 s and 4 ml of the old suspension transferred to 50 ml fresh BCD medium every 7 days. *Physcomitrella* gametophores were cultivated on solid BCD medium in Petri dishes and subcultured every 3 months. The cultures were kept at 25 °C under continuous light and suspension cultures were incubated on a gyratory shaker (100 rpm). For the transformation of *Physcomitrella patens* (see below), protonema tissue was incubated on solid BCD medium supplemented with 5 mM diammonium tartrate (BCDA), covered with cellophane, for 6 days (Cove et al. 2009a).

### Preparation of cDNA and amplification of a partial *AaC4H* sequence

RNA isolation was performed according to Chomczynski and Sacchi (1987). After checking the RNA integrity electrophoretically, cDNA was synthesized with the RevertAid^™^ First-Strand cDNA Synthesis Kit (Fermentas). Internal PCR primers were designed based on scaffold 11181 for C4H [Szövenyi, personal communication; Szövényi et al. (2015)] and synthesized by Eurofins Genomics (Suppl. Table S1). PCR assays of 25 µl were performed with up to 0.2 µg cDNA, 0.5 µl 10 mM dNTP mix, 0.5 µl of each primer (AaC4H_f, AaC4H_r, 100 µM), 3.0 µl 25 mM MgCl_2_, 5.0 µl 5 × GoTaq buffer and 0.1 µl GoTaq polymerase (5 U/µl, Promega) using the following program: 1 cycle 94 °C 120 s, 52–60 °C 60 s, 70 °C 90 s; 38 cycles 94 °C 30 s, 52–60 °C 60 s, 70 °C 90 s; 1 cycle 94 °C 120 s, 52–60 °C 60 s, 70 °C 600 s. Successful amplification of the target sequence was ensured by gel electrophoresis on a 0.7% agarose gel in TAE buffer (40 mM Tris, 20 mM acetic acid, 1 mM EDTA) using the GeneRuler DNA Ladder Mix (ThermoFisher) as marker. The PCR product was isolated with the NucleoSpin Gel and PCR Clean-up Kit (Macherey–Nagel) and the sequence was determined (Seqlab) after ligation into pDrive (Qiagen), transformation and multiplication in *E. coli* EZ (Qiagen).

### RACE-PCR and amplification of a full-length *AaC4H* sequence

RACE-PCR cDNA synthesis and RACE-PCR were conducted using the SMARTer^®^ RACE 5′/3′ kit (Takara/Clontech) with RACE primers (AaC4H_5′R, AaC4H_3′R; Suppl. Table S1) designed according to the sequence determined in the previous step. After isolation of the PCR products (NucleoSpin Gel and PCR Clean-up Kit, Macherey–Nagel) and ligation into the pRACE vector (Takara/Clontech), *E. coli* EZ were transformed and grown overnight. The plasmid was isolated and the sequence determined (Seqlab). For amplification of the full-length sequence of *AaC4H*, primers with restriction sites (underlined) were designed for SalI in the forward and EcoRI in the reverse primer ((AaC4H_fl_SalI_f and AaC4H_fl_EcoRI_r; Suppl. Table S1) for the integration into the entry vector pENTR^™^1A (Invitrogen). To be able to identify the protein later, a sequence encoding six histidine residues was added in front of the stop codon. PCR assays of 25 µl were performed as above but using Phusion^®^ High-Fidelity DNA Polymerase (2 U/µl; NEB) and buffer (NEB) with the following program: 1 cycle 94 °C 120 s, 60 °C 60 s, 70 °C 90 s; 38 cycles 94 °C 30 s, 60 °C 60 s, 70 °C 90 s; 1 cycle 94 °C 120 s, 60 °C 60 s, 70 °C 600 s. *E. coli* EZ cells were transformed with the purified PCR product ligated into pDrive for plasmid isolation and sequence determination (Seqlab). The full-length cDNA sequence has been entered into Genbank under the accession number MK778366.

### Construction of plasmids for the transformation of *Physcomitrella patens* with *AaC4H*

The *AaC4H* full-length sequence with *C*-terminal 6xHis codons was integrated into the entry vector pENTR^™^1A (Invitrogen) into the restriction sites for SalI and EcoRI. The LR recombination reaction was then performed with the Invitrogen kit following the manufacturer’s protocol. pTHUbiGate (kindly provided by Prof. Dr. S. Rensing, Philipps-Universität Marburg) served as the destination vector. The transgene is expressed under the control of the maize ubiquitin promotor (Perroud et al. 2011). *E. coli* DH5α was transformed with the reaction mixture and the plasmid replicated. The sequence was checked once again. For the transformation of *Physcomitrella patens*, the isolated plasmid was linearized with the restriction enzyme SwaI (NEB). After gel purification, contaminants were removed by ethanol precipitation (Crouse and Amorese 1987).

### Protoplastation and transformation of *Physcomitrella patens*

All media and methods for protoplastation and transformation were used as described in Cove et al. (2009a, b, c) with slight modifications. Six-day-old moss protonema tissue grown on cellophane disks placed on six BCDA medium Petri dishes was harvested and transferred to 15 ml filter-sterilized 2% Driselase (Sigma-Aldrich) in 8.5% mannitol (w/v). The protoplastation reaction was incubated for 3 h at room temperature with occasional gentle swirling. All centrifugation steps were performed at 100 g in a swinging bucket rotor. The protoplasts were filtered through 100-µm and 50-µm sieves, counted (Fuchs-Rosenthal counting chamber) and appr. 5 – 10 × 10^5^ protoplasts were used per transformation. After transformation, the cells were plated on solid PRMB medium covered with cellophane. The cellophane, carrying the regenerating protoplasts, was transferred after 1 week to BCDA medium containing 25 mg/l hygromycin B. After an additional week, the cellophane disks were transferred to antibiotic-free BCDA medium and incubated for 2 weeks. At last, the cellophane was again placed on a hygromycin B-containing BCDA medium for a week. Stable transformants were kept on solid BCD medium in Petri dishes. The plates were incubated at 25 °C in continuous light.

### Expression in *Physcomitrella patens* and protein isolation

Stable transformants were transferred from solid BCD medium to 50 ml liquid BCD in a 250-ml Erlenmeyer flask and maintained as described above. For expression, 10 ml of a 7-day-old suspension culture (homogenized with a tissue blender) was transferred to 200 ml BCD in a 1-l Erlenmeyer flask. The cells were kept on a gyratory shaker (100 rpm) at 25 °C under continuous light for 12 days. Then the cells were harvested by filtration and homogenized in a pre-cooled mortar together with 20% (w/w) of the fresh weight (FW) Polyclar 10 and 6 ml per g FW buffer (0.1 M Tris–HCl pH 7.0, 1 mM dithiothreitol, 1 mM diethyldithiocarbamate). The homogenate was centrifuged at 5000 g for 20 min at 4 °C. All attempts to isolate highly active microsomes resulted in reduction of the total enzyme activity although different preparation methods were used (Urban et al. 1994; Pompon et al. 1996; Abas and Luschnig 2010). Therefore, crude protein extracts were used for enzyme characterization.

All protein concentrations were determined according to Bradford (1976) using bovine serum albumin (1 mg/ml) as a standard.

### SDS-PAGE and Western blotting

Protein extracts were subjected to SDS-PAGE, which was essentially carried out according to Laemmli (1970). After SDS-PAGE, Western blotting was performed basically as specified by Mahmood and Yang (2012), but using the Towbin et al. (1979) buffer system. The expressed protein was detected with an anti-6x-His-tag monoclonal antibody (ThermoFisher MA1-21315). Goat anti-mouse IgG-Fc conjugated to alkaline phosphatase (Life Technologies, A16087) was used as secondary antibody. Colour reaction was obtained with nitroblue tetrazolium chloride/5-bromo-4-chloro-3-indolyl-phosphate according to the standard protocol on https://www.sysy.com/protocols/blot.php.

### Standard assay for AaC4H and reaction kinetics

Standard assays (in 1.5-ml reaction vials) contained 100 µl crude protein extract (0.1 mg protein), 7.5 µl 10 mM *t*-cinnamic acid (in 50% methanol), 12.5 µl 50 mM NADPH and 5 µl buffer (as above). Assays were mixed vigorously and incubated for 5 min at 25 °C under shaking at 1200 rpm in an Eppendorf Thermomixer. The reaction was stopped by the addition of 50 µl 6 N HCl. The assays were extracted twice with 500 µl ethyl acetate each and the ethyl acetate extracts combined and evaporated. The residues were redissolved in 50 µl MeOH with 0.01% H_3_PO_4_ (85%) and centrifuged after the addition of 50 µl aqueous 0.01% H_3_PO_4_. Quantification and analysis of the reaction product were performed by HPLC using a Hypersil ODS column (250 × 4 mm; pre-column: 20 × 4 mm; particle size 5 µm) and isocratic elution with 45% aqueous methanol containing 0.01% H_3_PO_4_ at a flow rate of 1 ml/min and detection at 309 nm. The reaction product 4-coumaric acid was quantified using a calibration curve of different concentrations of authentic 4-coumaric acid. The kinetic analysis data were obtained from four independent protein isolations with three technical replicates for each substrate concentration. Data were analysed with the GraphPad Prism 5 software using Michaelis–Menten, Lineweaver–Burk (not shown) and Hanes–Woolf models. The standard deviation (SD) was calculated from the mean values of each biological replicate.

### Construction of phylogenetic trees

For phylogenetic analysis, the translated amino acid sequence of AaC4H was aligned with other C4Hs from different species using the maximum likelihood method of the MEGA X software package. The robustness of the branch structure was evaluated with a bootstrap analysis (1000 replicates). The sequences for the phylogenetic tree were accessed from the BRENDA enzyme database and Renault et al. (2017) (see Suppl. Fig. S1 for accession numbers).

### Analysis of total phenolics in suspension-cultured thalli of Pp_AaC4H and Pp_WT

Over 21 days, *Physcomitrella patens* wildtype (Pp_WT) and transformed with *AaC4H* (Pp_AaC4H) were cultivated in 50 ml BCD medium at 25 °C under continuous light on a gyratory shaker (100 rpm). Tissue samples were collected every 7 days and stored at − 80 °C. After the addition of 100 µl 70% ethanol per 20 mg fresh weight, the suspension was mixed and incubated twice for 10 min at 80 °C in an ultrasonic bath. The samples were centrifuged for 10 min at 13,000*g*. To determine the total content of phenolic compounds, 25 µl of the supernatant was mixed with 475 µl water. 250 µl Folin–Ciocalteu reagent (Merck) was added and incubated for 15 min at room temperature. Then 2.5 ml alkaline reagent (0.1 N NaOH, 2% Na_2_CO_3_) was added and again incubated for 15 min at room temperature. The absorbance was measured photometrically at 760 nm (Jennings 1981). To calculate the content of phenolics, a calibration curve with caffeic acid was used. For this, 25 µl of different caffeic acid solutions (0, 0.25, 0.375, 0.5, 0.75 and 1 mg/ml in 70% ethanol) were used instead of the plant extract.

### Quantitative real-time PCR

Over 21 days, *Physcomitrella patens* transformed with *AaC4H* was cultivated in 50 ml BCD medium at 25 °C under continuous light on a gyratory shaker (100 rpm). Tissue samples were collected every second or third day and stored at − 80 °C. RNA extraction was done twice for each sample according to Chomczynski and Sacchi (1987). For the removal of DNA contaminants, 5 µg RNA was incubated with DNase (Thermo Scientific) using the manufacturer’s protocol and the remaining RNA was extracted with phenol/chloroform (Chomczynski and Sacchi 1987). 0.5 µg RNA was reverse-transcribed using the qScript™ cDNA SuperMix kit (Quanta). cDNA synthesis was performed twice for the DNase-digested RNA samples with higher integrity (*A*_260_/*A*_280_) and once for the RNA samples with lower integrity to account for biological variation. All RNA and cDNA samples were stored at − 20 °C until use.

Primers for AaC4H, a putative *Physcomitrella patens* C4H (PpC4H_1 = Pp3c25_10190V3.1) and an already identified *P. patens* C4H (PpC4H_2 = Pp3c4_21680V3.1 Renault et al. (2017)) were designed to obtain fragments with sizes of 212–219 bp. These fragments were all checked by sequencing. Serine threonine protein phosphatase 2a regulatory subunit (St-P 2a), involved in the regulation of signaling processes, was used as a reference gene. Other tested reference genes (actin 5, ubiquitin-conjugating enzyme E2 and elongation factor 1a) either showed an unspecific signal after agarose gel electrophoresis or more than one fragment were detected in the melting curve (data not shown) (Le Bail et al. 2013).

RT-qPCR was performed in a 96-well thermocycler (PikoReal96, Thermo Scientific) with the PerfeCTa SYBR Green SuperMix (Quanta) using the following program: 95 °C for 2 min, 50 cycles 95 °C 15 s, 52 °C 45 s, 68 °C 60 s. Each reaction consisted of 5 µl cDNA/water and 6.5 µl 2 × PerfeCTa SYBR Green SuperMix in a volume of 13 µl. cDNA serially diluted to concentrations ranging from non-diluted to 1:256 was used as a quantification standard to test amplification efficiency. Primers were added in concentrations of 192 nM for PpC4H_1 (*E* = 2.054) and PpC4H_2 (*E* = 2.098) and 385 nM for AaC4H (*E* = 1.959) and St-P 2a (*E* = 1.902). Replica of every isolated time point was measured twice on a 96-well plate and all measurements were repeated twice to account for technical variation. H_2_O was used as a negative control instead of cDNA. Data for the reference gene were acquired simultaneously in every run. Specific amplification of single fragments of all genes was confirmed by recording a melting curve by heating each PCR product from 50 to 95 °C.

Cq values (Suppl. Table S3) indicated the level of gene expression of each candidate gene for all samples. By comparing with St-P 2a, average *Δ*Cq values were generated and average values of day 0 were used to calculate fold-change of expression for each gene using the method described by Pfaffl (2001). Moreover, the relative expression in comparison to PpC4H_1 was calculated since this was the gene with the lowest overall expression. This ratio was calculated according to the following equation: ratio = E_St-P 2a_^Cq^^day^^X^/ E_C4H_^Cq day^^X^. For better comparability, PpC4H_1 was set to 1.

SD was generated from the mean values of at least six measured duplicates. SEM was calculated from the SD divided through the root of measured duplicates.

## Results

### Isolation of a cDNA encoding C4H from *Anthoceros agrestis*

Based on PCR primers directed against a partial putative *C4H* sequence from *Anthoceros agrestis* (scaffold 11181; Szövenyi, personal communication), an internal 657 bp fragment was isolated and sequenced. This fragment showed high similarities to other plant *C4H*s. 3′- and 5′-RACE PCR were used to amplify the cDNA ends. The full open reading frame consisted of 1578 bp encoding an amino acid sequence of 525 amino acid residues with a calculated molecular mass of 59.13 kDa (including 6xHis: 531 aa/59.95 kDa). The *C4H* sequence from *Anthoceros agrestis* has been classified as CYP73A260 (David Nelson, personal communication) and was deposited in GenBank under the accession number MK778366. As depicted in Suppl. Fig. S2, the amino acid sequence of AaC4H showed elements generally found in the canonic P450 monooxygenases such as the proline-rich region (Werck-Reichhart et al. 2002), the PERF motif, the heme-binding cysteine motif, and the threonine-containing binding pocket motif (Schuler 1996; Mizutani et al. 1997; Chapple 1998). The AaC4H amino acid sequence showed high identities (> 75%) to predicted or characterized C4Hs from higher plants (protein BLAST). A phylogenetic analysis of C4H amino acid sequences (Suppl. Fig. S1), analysed with the maximum likelihood algorithm of the MEGA X program package, supports the hypothesis that hornworts may be the youngest group of the bryophytes. The tree shows two branches, the first with liverworts and mosses and the second with hornworts, ferns, lycophytes, gymnosperms, and angiosperms. It indicates that AaC4H is more similar to C4Hs from ferns and lycophytes than to the respective enzymes from mosses and liverworts.

### Expression of *Anthoceros agrestis* C4H in *Physcomitrella patens* and characterization of C4H activities

**Fig. 1:**
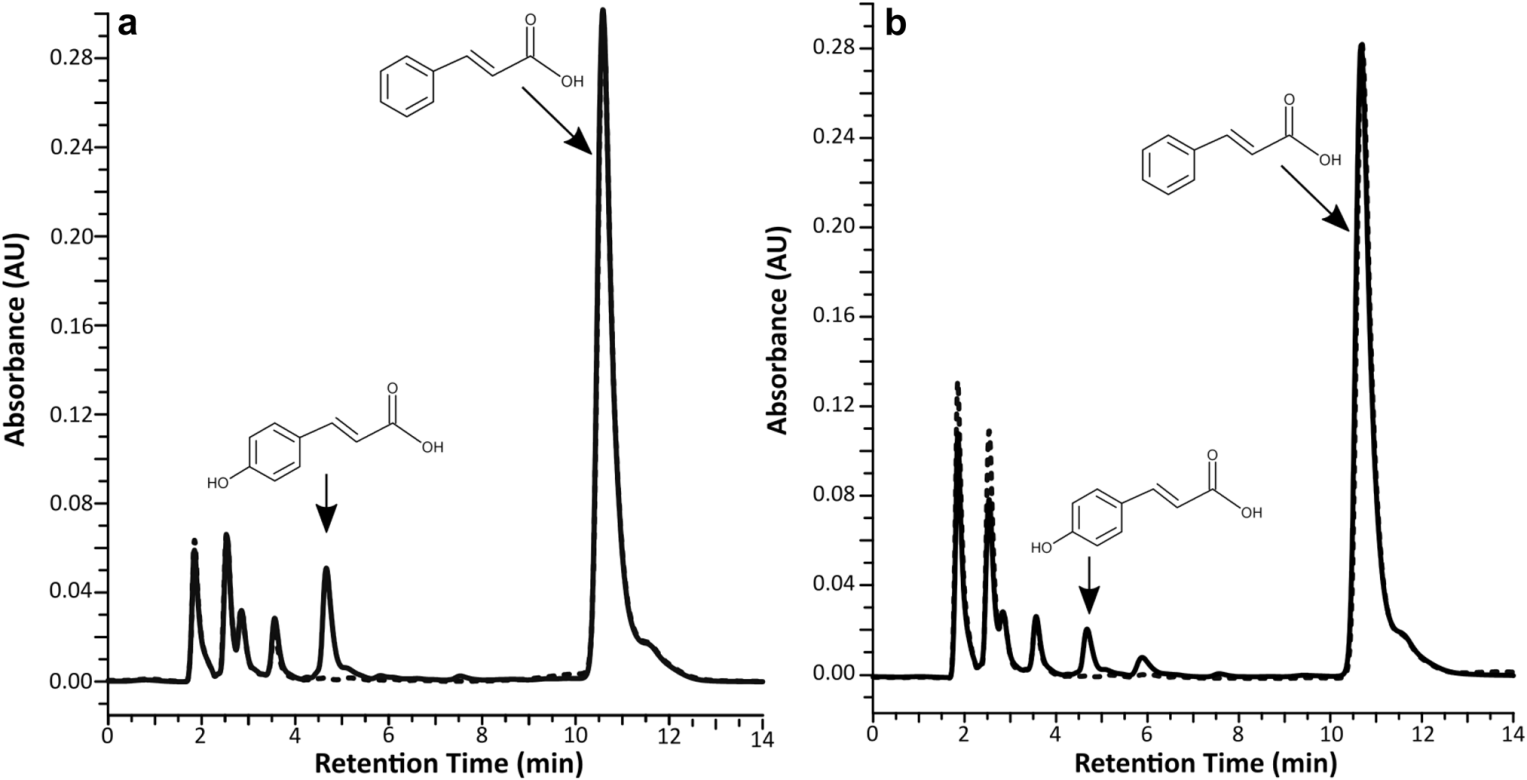
Reaction of C4H with *t*-cinnamic acid analysed by HPLC at 309 nm (isocratic elution with 45% methanol/0.01% H_3_PO_4_). **a** HPLC-chromatogram of Pp_AaC4H after 0 min (dashed line) and 30 min (solid line) reaction time. **b** HPLC-chromatogram of Pp_WT after 0 min (dashed line) and 30 min (solid line) reaction time

After transformation of *Physcomitrella patens* protoplasts and regeneration, three stable Pp_AaC4H transformants were obtained. One transformant (Pp_AaC4H 1) showed a band of the correct size (~ 60 kDa) after Western blotting. Supplementary Figure S3 demonstrates that AaC4H is localized in the microsomal fraction. All enzyme activity assays were performed in parallel with the wild type (Pp_WT). Since *Physcomitrella* itself has C4H activities stemming from six putative C4H genes (JGI Phytozome 12 (https://phytozome.jgi.doe.gov/pz/portal.html); Suppl. Table S2) gene knockout of all six genes was not considered feasible. As C4H activity was strongly reduced after the preparation of microsomes (also in the untransformed culture), crude protein extracts were used for further activity assays. After HPLC analysis of C4H activity assays, those made with protein extracts from transformed cultures revealed the formation of at least double to triple the amount of 4-coumaric acid than the wild-type control (Fig. 1). 3-Hydroxy- and 4-hydroxycinnamic acids were also tested as substrates, but were only scarcely converted to caffeic acid (0.7% and 2.5% related to cinnamic acid as 100%, respectively). Since this formation of caffeic acid was in the same range in Pp_WT and Pp_AaC4H assays, it was mainly attributed to activities already present in *Physcomitrella patens* wildtype. Enzyme assays with benzoic acid as alternative putative substrate showed no conversion to 4-hydroxybenzoic acid. Using NADH as cosubstrate resulted in a conversion rate of approximately 20% in comparison to the assay with the same concentration of NADPH as cosubstrate (Suppl. Fig. S4).

The pH and temperature optima for C4H from Pp_AaC4H and Pp_WT were determined to be around pH 7.0 and 25 °C, respectively (Suppl. Fig. S5).

**Fig. 2:**
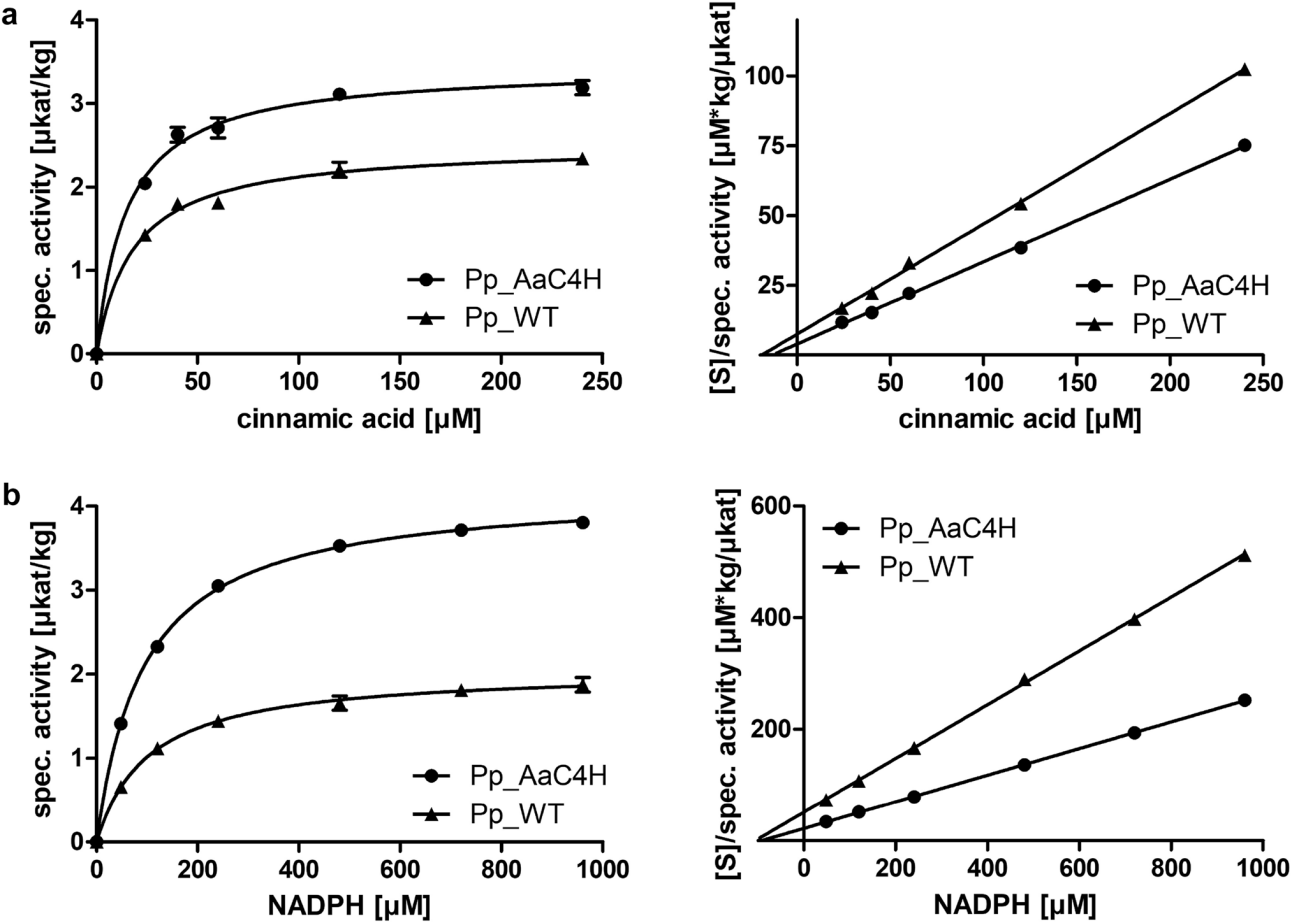
Dependence of C4H on cinnamic acid and NADPH. **a** Michaelis–Menten (left) and Hanes–Woolf diagrams (right) for *t*-cinnamic acid. **b** Michaelis–Menten (left) and Hanes–Woolf diagrams (right) for NADPH. Shown are representative graphs from one biological replicate with three repetitions; error bars show SD

For the determination of kinetic data for AaC4H and PpC4Hs, assays with cinnamic acid in concentrations up to 240 µM with 5 mM NADPH were performed with 5 min reaction time to ensure the determination of initial reaction velocities. The resulting substrate saturation curves for cinnamic acid led to slightly different apparent *K*_m_ values (± SD; as determined from Michaelis–Menten curves) of 17.3 ± 5.0 µM for Pp_AaC4H and 25.1 ± 8.9 µM for Pp_WT (Fig. 2).

To determine the K_m_ value for NADPH, enzyme assays with 400 µM cinnamic acid were incubated for 5 min with up to 960 µM NADPH. The *K*_m_ values (± SD; as determined from Michaelis–Menten curves) were approximately the same in C4H assays of Pp_AaC4H (88.0 ± 19.5 µM) and Pp_WT (92.3 ± 10.8 µM) (Fig. 2), which is due to the fact that both reactions are supplied with electrons by the same *Physcomitrella patens* NADPH:cytochrome P450 reductase(s) (CPR or POR).

### Gene expression analysis and total phenolic content

**Fig. 3:**
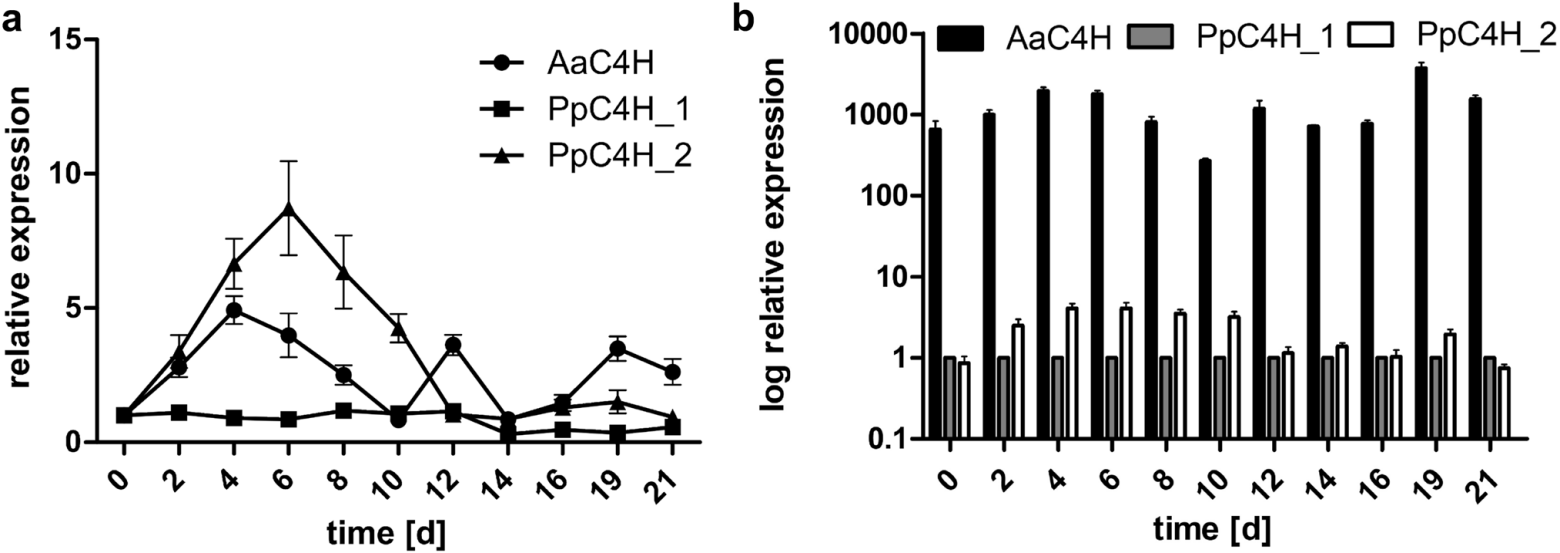
Quantitative real-time PCR analysis of Pp_AaC4H cinnamic acid 4-hydroxylases AaC4H, PpC4H_1, and PpC4H_2. St-P 2a was used as the reference gene. Each data point represents the mean of at least six measured duplicates from two different RNAs, the error bars represent the standard error. **a** Time-dependent expression analysis calculated with the method described by Pfaffl (2001). **b** Expression (logarithmic scale) in relation to PpC4H_1. Relative expression was calculated using the formula: ratio = E_St-P__2a_^Cq^^day^^X^/E_C4H_^Cq^^day^^X^and PpC4H_1 was set to 1

Total RNA from transformant Pp_AaC4H was extracted after taking samples every second or third day over 21 days cultivation time and gene expression levels were measured by quantitative real-time PCR (for mean Cq values of each measured duplicate see Suppl. Table S3) using ST-P 2a as reference gene. For expression analysis, the two most highly expressed PpC4H genes (Phytozome (https://phytozome.jgi.doe.gov/pz/portal.html): PpC4H_1 = Pp3c25_10190V3.1 and PpC4H_2 = Pp3c4_21680V3.1; Suppl. Table S2) under the used cultivation conditions were chosen. Expression of PpC4H_1 stayed roughly steady over the first 12 days and was reduced to about half during the late cultivation time. Compared to day 0, PpC4H_2 had the highest increase in expression reaching its maximum after 6 days with a ninefold increase. Afterwards, gene expression of PpC4H_2 dropped and reached expression levels similar to day 0. AaC4H had its highest expression rate on day 4 with fivefold compared to day 0 and slightly increased expression rates (appr. 3.5×) on days 12 and 19 (Fig. 3a).

Related to the expression of the house-keeping gene St-P 2a, PpC4H_1 had the lowest expression rate. To clarify this further, the expression of PpC4H_1 was set to 1 for each culture day and the expression levels of PpC4H_2 and AaC4H related to this expression. After 4–6 days, PpC4H_2 showed fourfold expression compared to PpC4H_1 and after 12 days they both reached similar levels. Compared to both genes, AaC4H was expressed at much higher rates. Starting with a 650-fold expression on day 0 (as compared to PpC4H_1), the rate increased to a around 2000-fold and 3700-fold after 4 and 19 days, respectively. The lowest expression level was observed after 10 days with a 270-fold expression compared to PpC4H_1 (Fig. 3b).

To investigate the impact of the high expression rates of AaC4H in *Physcomitrella patens*, the total content of phenolic compounds was determined. However, this content barely changed over 21 days. The amount in the suspension-cultured *Physcomitrella patens* thalli Pp_AaC4H and Pp_WT always was appr. 650–950 µg/g fresh weight (Suppl. Fig. S6).

## Discussion

C4Hs, although being the best investigated cytochrome P450 monooxygenases from plants, have only rarely been described from lower plants on the molecular level. Only three liverwort genes encoding C4H from *Marchantia paleacea* and *Plagiochasma appendiculatum* have been isolated and functionally expressed in yeast and characterized in yeast (Liu et al. 2017). C4H from the suspension-cultured hornwort *Anthoceros agrestis* has been characterized biochemically by Petersen (2003). We here report the isolation of the *C4H* cDNA from the same hornwort as well as the heterologous expression of *AaC4H* in the moss *Physcomitrella patens*. Comparing AaC4H amino acid sequences with corresponding sequences from other plants in a phylogenetic analysis suggests the existence of two major clades, the first comprising the liverworts and mosses, and the second encompassing sequences from hornworts, ferns, lycophytes, gymnosperms and angiosperms. Current research shares the opinion that liverworts and mosses form a clade (Wickett et al. 2014; Ruhfel et al. 2014). This was supported in our analysis. The liverworts are often regarded as the phylogenetically oldest clade of the bryophytes, followed by the mosses and the hornworts, where the hornworts represent the sister group to vascular plants (Qiu et al. 2006; Ligrone et al. 2012; Ruhfel et al. 2014). This, however, is currently under discussion (Szövenyi et al. 2015). The placement was generally confirmed in the phylogenetic analysis of C4H amino acid sequences, although it also suggests that C4Hs from gymnosperms and angiosperms might have evolved concurrently. Renault et al. (2017) analysed CYP73 sequences from higher and lower plants and resolved two classes, both containing angiosperms as well as gymnosperms, and a separate group encompassing non-seed plants.

*Physcomitrella patens* as a novel expression system has already been used for the heterologous expression of enzymes involved in plant specialized metabolism (Anterola et al. 2009; Bach et al. 2014; Zhan et al. 2014; Pan et al. 2015; Khairul Ikram et al. 2017). Stable transformants can be obtained due to the high rates of homologous recombination making the permanent use of selective media unnecessary (Schaefer et al. 1991). Because of its close relationship and similar gene structure, similar GC content, the ability of posttranslational modifications and the presence of own CPR genes, it was chosen as the expression system for AaC4H using the *Physcomitrella patens* CPR(s) as electron transferring enzyme(s). The expression in *Physcomitrella*, however, has the disadvantage that transformation and expression are time-consuming. Besides, the moss has own putative *C4H* genes in addition to four putative *CPR* genes (see Suppl. Table S2). Gene deletion of up to six *Physcomitrella patens* CYP73A genes seemed unfeasible; therefore, all experiments were made with the Pp_AaC4H transformant and the *Physcomitrella* wildtype in parallel. One stable transformant showing AaC4H protein formation was further characterized. This transformant produced considerably more 4-coumaric acid in in vitro assays compared to wildtype showing the impact of the introduced C4H from *Anthoceros agrestis*. To obtain higher enzyme activities all C4H assays were performed with crude protein extracts instead of microsome preparations. Although different methods were used to extract the membrane fraction (Urban et al. 1994; Pompon et al. 1996; Abas and Luschnig 2010), the total 4-coumaric acid production was always reduced to appr. 25% compared to assays using the crude protein extract. This effect was also observed using cells from a suspension culture of *Anthoceros agrestis* with a loss of activity of appr. 60% after microsome preparation (data not shown). It has not been investigated whether this is a general problem or specific for bryophyte enzymes. Since microsomes are commonly used in work with P450s (e.g., Sullivan and Zarnowski 2010; Liu et al. 2017; Renault et al. 2017), this problem seems yet to be unnoticed. The optimum pH for C4H from both the transformed and the wildtype culture is in the range 7.0–7.5 which is regarded as typical for C4H (Werck-Reichhart 1995) and the majority of other cytochrome P450 enzymes from higher plants. The temperature optimum of the C4H reaction is generally in the range of 20–30 °C, which is also the case for Pp_AaC4H and Pp_WT with 25 °C. Both values of the heterologously expressed AaC4H correspond to the results for C4H measured in microsome preparations from *Anthoceros agrestis* suspension cultures (Petersen 2003). The corresponding values for C4H from the liverworts *Plagiochasma appendiculatum* and *Marchantia paleacea* were at 30 °C and pH 7.0 (Liu et al. 2017). Werck-Reichhart (1995) reported that the apparent *K*_m_ for cinnamic acid is in the range of 2–30 µM for most C4Hs. In our experiments, the *K*_m_ for cinnamic acid was at 17.3 ± 5.0 µM for Pp_AaC4H and 25.1 ± 8.9 µM for Pp_WT showing the slightly higher affinity of AaC4H for cinnamic acid than the PpC4H(s). This is in agreement with the measured *K*_m_ value of 5 µM for C4H from *Anthoceros agrestis* suspension cultures (Petersen 2003). It is unknown to date whether *Anthoceros agrestis* also has more than one gene encoding C4H. The *K*_m_ values for cinnamic acid for the three heterologously synthesized (in yeast) C4H genes from liverworts ranged between 0.7 and 1.7 µM (Liu et al. 2017). These liverwort C4Hs also had some activity towards 3-hydroxycinnamic acid resulting in the formation of caffeic acid. In our assays, we could not detect significant activity of AaC4H with 3-hydroxy- and 4-hydroxycinnamic acids. The observed formation of caffeic acid from these substrates was attributed to the activity of the *Physcomitrella patens* wildtype protein extract.

NADPH is the preferred electron donor for the majority of cytochrome P450 enzymes. Using NADH instead in our experiments, resulted in about 20% product formation compared with NADPH. NADH as a comparatively potent electon donor has been reported for CPR from *Anthoceros agrestis* (isolated from suspension cultures) where 50% of the activity with NADPH was achieved with the same concentration of NADH (Petersen 2003). Whether this might be a feature typical for CPR from lower plants can only be shown with the characterization of more lower plant CPRs. The *K*_m_ values for NADPH for both, Pp_AaC4H (88.0 ± 19.5 µM) and Pp_WT (92.3 ± 10.8 µM) were similar, which was expected since the PpCPR provides the electrons also for AaC4H. This indicates that the additional AaC4H can freely work with PpCPR and has no negative impact on the P450 redox partner.

Tissue samples of Pp_AaC4H were collected over 3 weeks for quantitative real-time PCR measurements targeting AaC4H, an already identified *P. patens* C4H (PpC4H_2; Renault et al. 2017) and a second putative *Physcomitrella* C4H (PpC4H_1) as well as the reference gene St-P 2a (used for normalization). While PpC4H_1 was constantly expressed at a low level, PpC4H_2 and AaC4H had similar expression patterns. Both had their maximum expression levels at days 4–6. The expression of PpC4H_2 decreased after 6 days and after 12 days the expression remained on the same level as on day 0. This corresponds to the expression data provided by Phytozome that PpC4H_2 expression is high in protonema cells and comparably low in the gametophore suggesting that after 12 days protonemata had developed into gametophore tissue. A closer look at the expression levels revealed that mRNA of AaC4H always was present at much higher rates, at least 270-fold compared to PpC4H_1. The highest difference was observed after 4 and 19 days. Here the expression was 2000- to 4000-fold. In comparison, PpC4H_2 mRNA was present fourfold compared to PpC4H_1. The difference between the two C4Hs of *Physcomitrella* is reflected in the data of Phytozome (https://phytozome.jgi.doe.gov/pz/portal.html, accessed 27 August 2019), where expression in different media is displayed. Also here, PpC4H_2 mRNA is often present in higher quantities than PpC4H_1. The extremely high expression levels of AaC4H in comparison to *Physcomitrella*’s own C4H reflect the effectiveness of the maize ubiquitin promotor even in a lower plant as described by Schaefer (2002). This result underlines *Physcomitrella* as a competitive expression organism. However, the very high expression levels of AaC4H compared to PpC4H are not reflected in enzyme activity data. Here the differences between wildtype and the AaC4H-transformed *Physcomitrella* were considerably lower. This could be explained either by low translation rates of the AaC4H mRNA or by the formation of non-functional AaC4H proteins or by the restriction of the electron transfer capacity of the *Physcomitrella* CPR. This might be overcome by increasing the expression rates of CPR—either from *Physcomitrella* itself or from *Anthoceros agrestis*—using strong promoters for these genes as well.

The increased C4H activity due to the transfer of AaC4H into *Physcomitrella patens*, however, barely changed the phenolic content. A similar effect was observed in transgenic tobacco plants (Sewalt et al. 1997; Blount et al. 2000). While down-regulation of C4H resulted in a reduced accumulation of caffeic acid esters, overexpression of C4H, on the other hand, did not result in an increased accumulation of phenolic compounds and lignin.

This work aimed at showing that functional expression of a gene encoding a membrane-bound protein in the *Physcomitrella patens* system is possible in addition to more frequently used expression systems like *Saccharomyces cerevisiae*. The foreign C4H from *Anthoceros agrestis* was characterized alongside C4H(s) from *Physcomitrella patens*.

## Conclusion

*Physcomitrella patens* was successfully transformed with the coding sequence of C4H from the hornwort *Anthoceros agrestis* by protoplast transformation and homologous recombination. This resulted in catalytically active C4H which could be characterized biochemically besides the own *Physcomitrella* C4H(s). This illustrates that *Physcomitrella patens* can be used as expression system for the production of active plant cytochrome P450 enzymes.

## Electronic supplementary material

Below is the link to the electronic supplementary material.

Supplementary file1 (PDF 1763 kb)
