# Supplementary material for: Functional expression and characterization of cinnamic acid 4-hydroxylase from the hornwort Anthoceros agrestis in Physcomitrella patens
Source: Plant Cell Rep. 2020 Feb 13;39(5):597–607. doi: 10.1007/s00299-020-02517-z (PMC7165133; doi:10.1007/s00299-020-02517-z)
Supplement: Supplementary file 1 — Supplementary file1 (PDF 1763 kb) [file 299_2020_2517_MOESM1_ESM.pdf]

Supplemental Material

Plant Molecular Biology

Julia Wohl, Maike Petersen*

Functional expression and characterization of cinnamic acid 4-hydroxylase from the hornwort *Anthoceros agrestis* in *Physcomitrella patens*

Institut für Pharmazeutische Biologie und Biotechnologie, Philipps-Universität Marburg, Robert-Koch-Str. 4, D-35037 Marburg, Germany

* Corresponding author: petersen@staff.uni-marburg.de; telephone +49 6421 2825821

Suppl. Table S1 List of PCR primers: binding sequence underlined, restriction sites marked red, His-Tag marked green

Primer name	Sequence
AaC4H_f	ATGGCTTCCGGTGAAACCAC
AaC4H_r	GACAAATAGGGGATCGTCCTCG
AaC4H_5´R	GATTACGCCAAGCTTGGTGAAGAAGGGCACAGTCATGATCC
AaC4H_3´R	GATTACGCCAAGCTTGCAACCTGTCGGACCTTGCGAAG
AaC4H_fl_EcoRI_f	GCATGAATTCATGGCTTCCGGTGAAACCAC
AaC4H_fl_NotI_r	GCATGCGGCCGCCATATCAGGGCGTGGCTTGA
AaC4H_fl_SalI_f	TTAGTCGACAACCATGGCTTCCGGTGAAACCACTCTTGGCAGC
AaC4H_fl_EcoRI_r	TATGAATTCTCAATGATGATGATGATGATGCATATCAGGGCGTGGC
AaC4H_qPCR_f	CTTCTGGACACAAAGCGCA
AaC4H_qPCR_r	CTCCCTCACTTTCTGCTG
PpC4H1_qPCR_f	GTTGTCAACCTTGGGACC
PpC4H1_qPCR_r	CTTCACGTATGCGGGTCT
PpC4H2_qPCR_f	GTTGAATAGCGTGAATCCTCC
PpC4H2_qPCR_r	CTCATTTCGAATCCTGGTTTGAAT
St-P 2a_qPCR_f	GTCTAGTTAGTCCTTTGGTCCT
St-P 2a_qPCR_r	GCCTATTTCTATAATGACTCCGT

Suppl. Table S2 Putative and identified cinnamate 4-hydroxylase and NADPH:cytochrome P450 reductase sequences extracted from the JGI Phytozome 12 database (https://phytozome.jgi.doe.gov/pz/portal.html#). The C4H identified by Renault et al. (2017) is underlined. The genes written in bold letters were included in gene expression analysis

**Cinnamate 4-hydroxylase (CYP73A)**	
Pp3v3_17840V3.1	Pp3c13_14870V3.1
**Pp3c4_21680V3.1**	Pp3c16_23740V3.1
Pp3c12_6560V3.1	**Pp3c25_10190V3.1**
**NADPH:cytochrome P450 reductase**	
Pp3c8_19940V3.1	Pp3c20_9680V3.1
Pp3c14_22890V3.1	Pp3c24_17560V3.1

Suppl. Table S3 Gene expression analysis by quantitative real time PCR for AaC4H and the *Physcomitrella patens* C4Hs PpC4H_1 = Pp3c25_10190V3.1 and PpC4H_2 = Pp3c4_21680V3.1 as well as ST-P 2a (serine threonine protein phosphatase 2a regulatory subunit) as housekeeping gene. Mean Cq value of each measured duplicate are listed

**St-P 2a**											
**day**	**0**	**2**	**4**	**6**	**8**	**10**	**12**	**14**	**16**	**19**	**21**
RNA_1_1.1	26.14	25.37	24.57	23.99	24.49	23.47	26.05	24.43	23.12	23.25	23.48
RNA_1_1.2	26.37	24.99	24.34	23.47	24.35	23.57	25.98	24.09	22.98	23.55	23.70
RNA_1_1.3	26.36	25.58	24.59	24.28	25.31	23.55	26.34	24.45	23.41	23.86	23.44
RNA_2_1	26.83	25.29	25.16	24.89	24.39	23.48	25.00	25.21	25.11	22.59	24.55
RNA_2_2	26.01	26.07	25.75	24.59	24.81	23.32	27.38	25.28	24.05	23.56	
RNA_2_3	25.70	25.80	24.80	25.03	24.40	23.92	26.24	25.16	24.04	23.34	23.86
RNA_1_2.1	27.47	25.67	24.53	24.53	24.85	22.34	24.74	24.41	23.83	22.79	24.05
RNA_1_2.2	27.14	25.91	24.69	24.88	24.42	23.62	25.71	24.56	24.75	23.22	24.69
**AaC4H**											
**day**	**0**	**2**	**4**	**6**	**8**	**10**	**12**	**14**	**16**	**19**	**21**
RNA_1_1.1	25.31	22.96	21.13	20.60	22.53	23.21	23.56	24.00	22.01	20.50	21.14
RNA_1_1.2	25.54	22.77	21.26	20.89	22.60	23.68	23.25	23.75	21.17	20.60	21.22
RNA_1_1.3	25.77	22.82	21.35	20.91	22.87	24.02	23.81	23.59	21.45	21.02	20.68
RNA_2_1	24.72	22.74	22.69	20.98	21.41	21.55		25.29	23.16	21.13	23.25
RNA_2_2	23.30	22.85	22.25	22.12	21.72	20.92	22.93	21.07	20.56	21.02	21.78
RNA_2_3	24.14	23.18	21.70	22.02	21.36	22.39	23.09	23.02	23.12	20.77	21.44
RNA_1_2.1	26.26	23.04	20.95	22.58	21.72	21.08	21.14	24.57	23.47	20.25	22.40
RNA_1_2.2	26.32	23.33	21.89	22.52	22.93	21.19	21.96	25.49	24.09	21.15	23.46
**PpC4H_1**											
**day**	**0**	**2**	**4**	**6**	**8**	**10**	**12**	**14**	**16**	**19**	**21**
RNA_1_1.1	30.94	30.33	29.86	29.05	29.67	29.57	32.88	31.45	29.22	29.69	29.83
RNA_1_1.2	32.04	30.65	30.14	29.60	30.44	30.06	31.72	31.47	29.95	30.82	30.35
RNA_1_1.3	32.45	31.14	30.04	30.10	30.44	30.13	31.26	31.09	29.51	30.15	29.54
RNA_2_1	32.88	32.60	32.24	30.38	31.03	30.11	29.38	30.99	30.69	30.22	30.44
RNA_2_2	31.61	29.47	31.42	31.38	30.12	28.91	33.15	32.56	31.20	31.25	31.15
RNA_2_3	31.53	31.75	31.01	30.74	29.40	28.93	31.62	32.61	31.17	31.53	30.48
RNA_1_2.1		31.46	31.25	29.51	30.26	27.28	28.63	32.20	31.41	31.16	30.59
RNA_1_2.2		30.86	29.13	29.85	29.30	27.27	29.45	32.89	31.49	30.79	30.32
**PpC4H_2**											
**day**	**0**	**2**	**4**	**6**	**8**	**10**	**12**	**14**	**16**	**19**	**21**
RNA_1_1.1	31.22	29.35	27.25	26.09	27.62	27.12	31.59	29.69	28.17	28.32	29.30
RNA_1_1.2	30.66	28.28	26.84	26.14	27.40	26.81	30.13	30.29	27.36	28.23	28.45
RNA_1_1.3	31.94	29.22	27.74	26.80	28.10	27.71	31.17	29.58	27.93	28.33	29.30
RNA_2_1	34.85	29.62	29.56	28.02	28.39	25.62	27.05	30.10	31.17	29.23	30.06
RNA_2_2	31.45	29.75	29.11	28.21	27.26	26.96		31.37	29.74	29.68	30.66
RNA_2_3	30.43	29.86	28.70	28.63	27.23	26.48	31.63	31.65	30.00	29.80	30.42
RNA_1_2.1	33.46	29.58	27.84	27.60	26.36	24.98	27.13	30.51	30.57	28.06	29.65
RNA_1_2.2	34.69	30.50	29.13	28.18	28.20	26.10	28.38	30.13	32.41	28.99	32.99

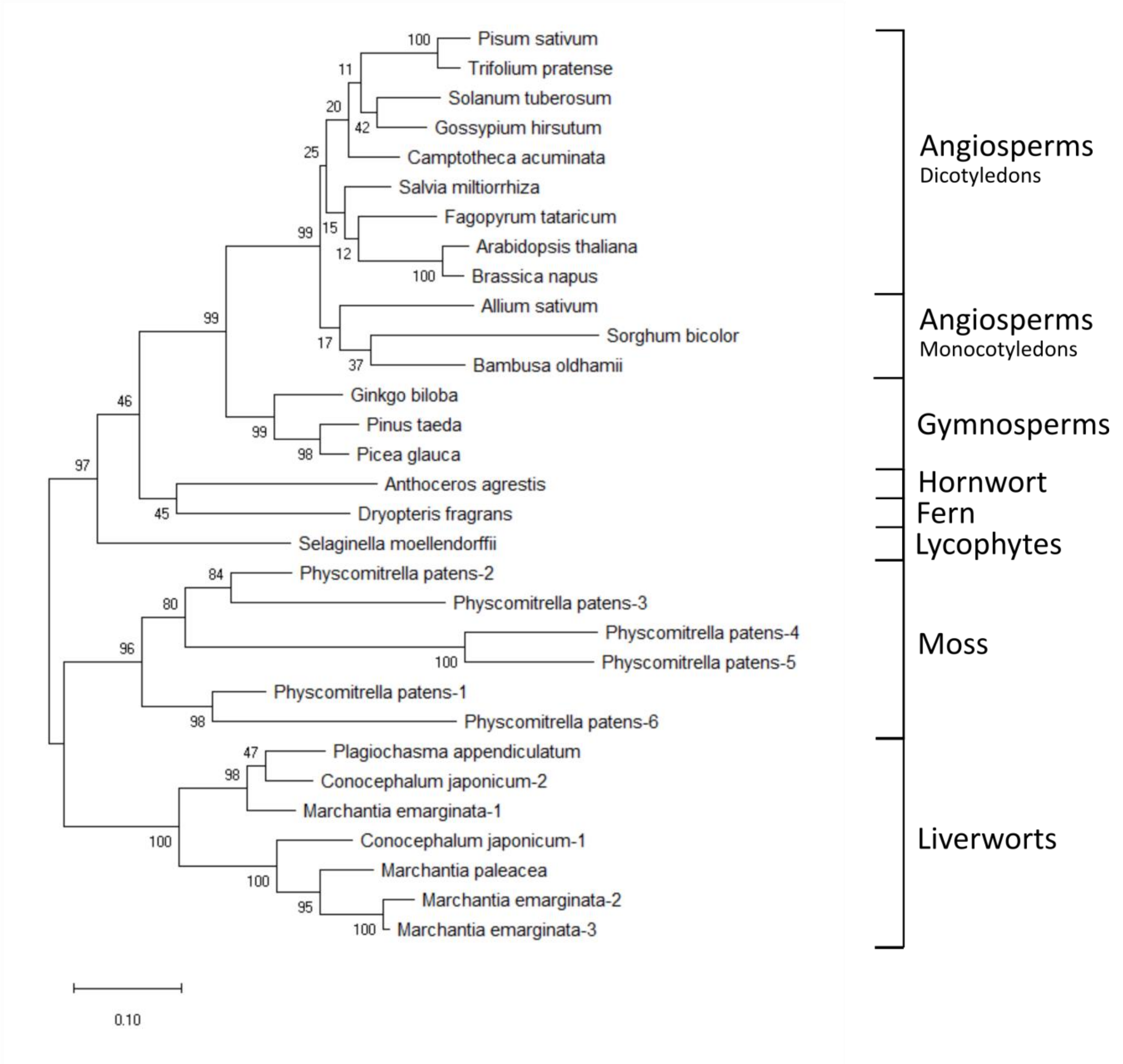

Suppl. Fig. S1 Phylogenetic analysis of C4H amino acid sequences from *Pisum sativum* (Q43067), *Trifolium pratense* (B2LSD9), *Solanum tuberosum* (Q2LAD8), *Gossypium hirsutum* (E4W6M8), *Camptotheca acuminata* (AAT39513), *Salvia miltiorrhiza* (A3FIN3), *Fagopyrum tataricum* (A0A2R2ZJB8), *Arabidopsis thaliana* (P92994), *Brassica napus* (A5GZU5), *Allium sativum* (ADO24190), *Sorghum bicolor* (Q94IP1), *Bambusa oldhamii* (D2K8K9), *Ginkgo biloba* (AAW70021), *Pinus taeda* (AAD23378*), Picea glauca (A0A0G7ZP02), Anthoceros agrestis* (MK778366), *Dryopteris fragrans* (AHI17493), *Selaginella moellendorffii (D8S0Y1), Physcomitrella patens*-2 (Pp3c4_21680V3.1), *Physcomitrella patens*-3 (Pp3c12_6560V3.1), *Physcomitrella patens*-4 (Pp3c13_14870V3.1), *Physcomitrella patens*-5 (Pp3c3_17840V3.1), *Physcomitrella patens*-1 (Pp3c25_10190V3.1), *Physcomitrella patens*-6 (Pp3c16_23740V3.1), *Plagiochasma appendiculatum* (A0A1Z2R7G8), *Conocephalum japonicum*-2 (A0A1Z2R7G6), *Marchantia emarginata*-1 (A0A1Z2R7H6), *Conocephalum japonicum*-1 (A0A1Z2R7H4), *Marchantia paleacea* (A0A1Z2R7G9), *Marchantia emarginata*-2 (A0A1Z2R7H5) and *Marchantia emarginata*-3 (A0A1Z2R7I0). The maximum likelihood tree was constructed using the MEGA X software. The robustness of the branch structure was evaluated with a bootstrap analysis (1000 replicates). Evolutionary distance is represented with the bars

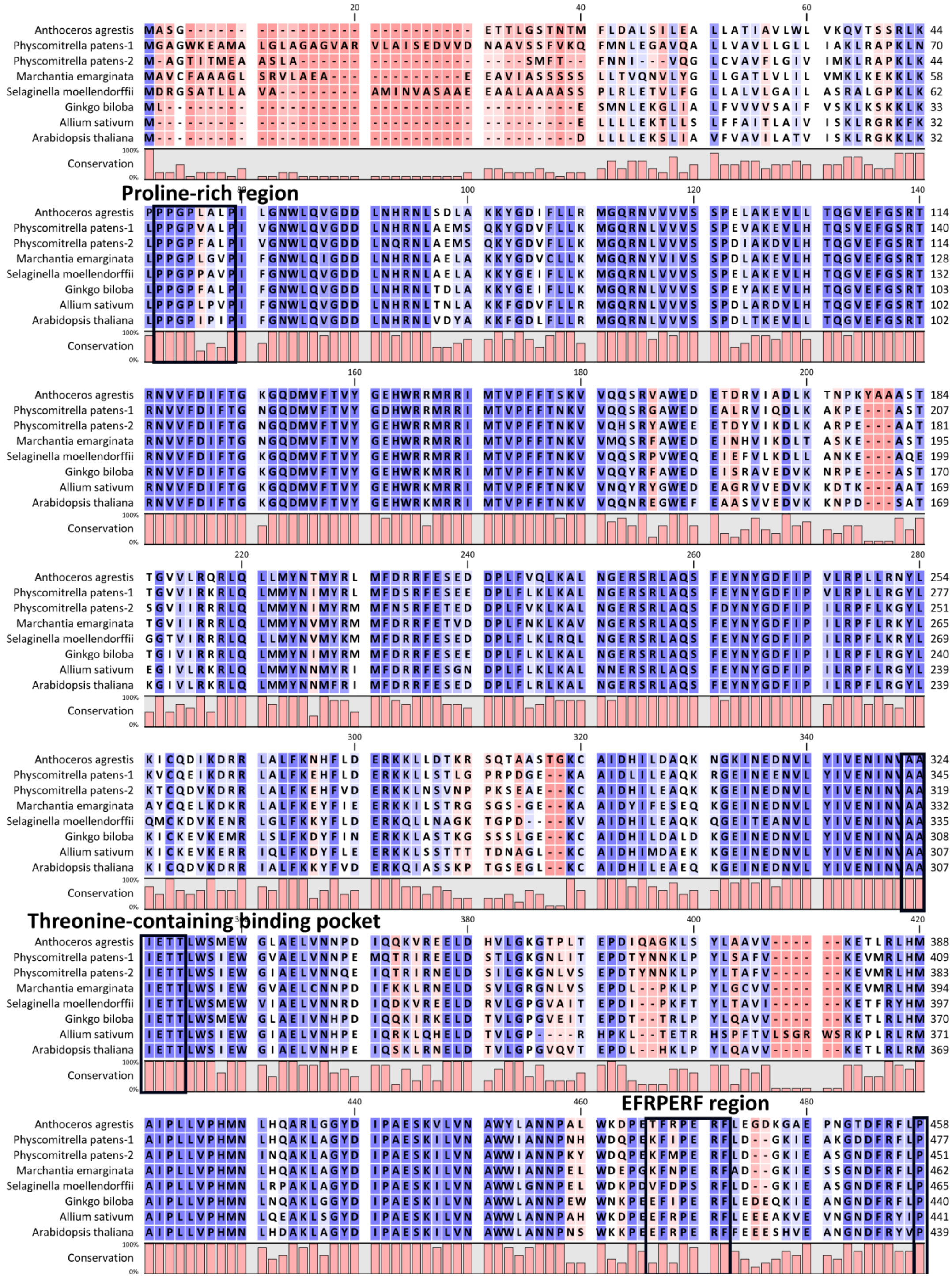

Suppl. Fig. S2 Alignment of amino acid sequences of C4H from *Anthoceros agrestis* (this publication), *Physcomitrella patens*-1 (Pp3c25_10190V3.1), *Physcomitrella patens*-2 (Pp3c4_21680V3.1), *Marchantia emarginata*-1 (A0A1Z2R7H6), *Selaginella moellendorffii* (D8S0Y1), *Ginkgo biloba* (AAW70021), *Allium sativum* (ADO24190) and *Arabidopsis thaliana* (P92994). Highly conserved amino acids are marked blue, low conservation is marked red. Typical conserved sequence motifs are marked by boxes

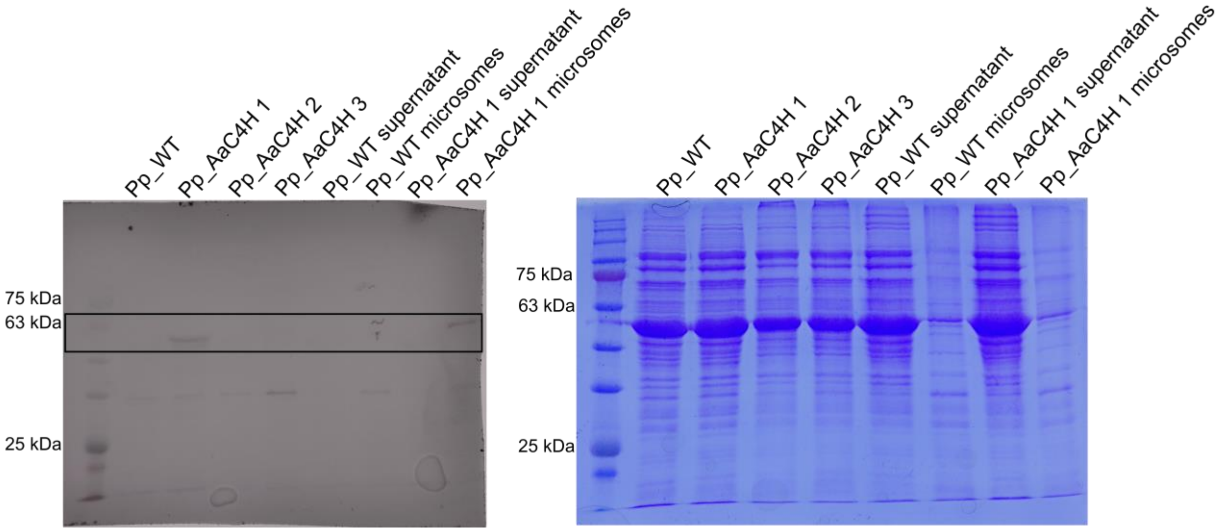

Suppl. Fig. S3 Western blot analysis (**left**) and Coomassie stained SDS-PAGE (**right**) after Western blotting of His-tagged AaC4H expressed in three different stable *Physcomitrella patens* transformants (Pp_AaCAH). A *Physcomitrella patens* wild-type culture (Pp_WT) served as negative control

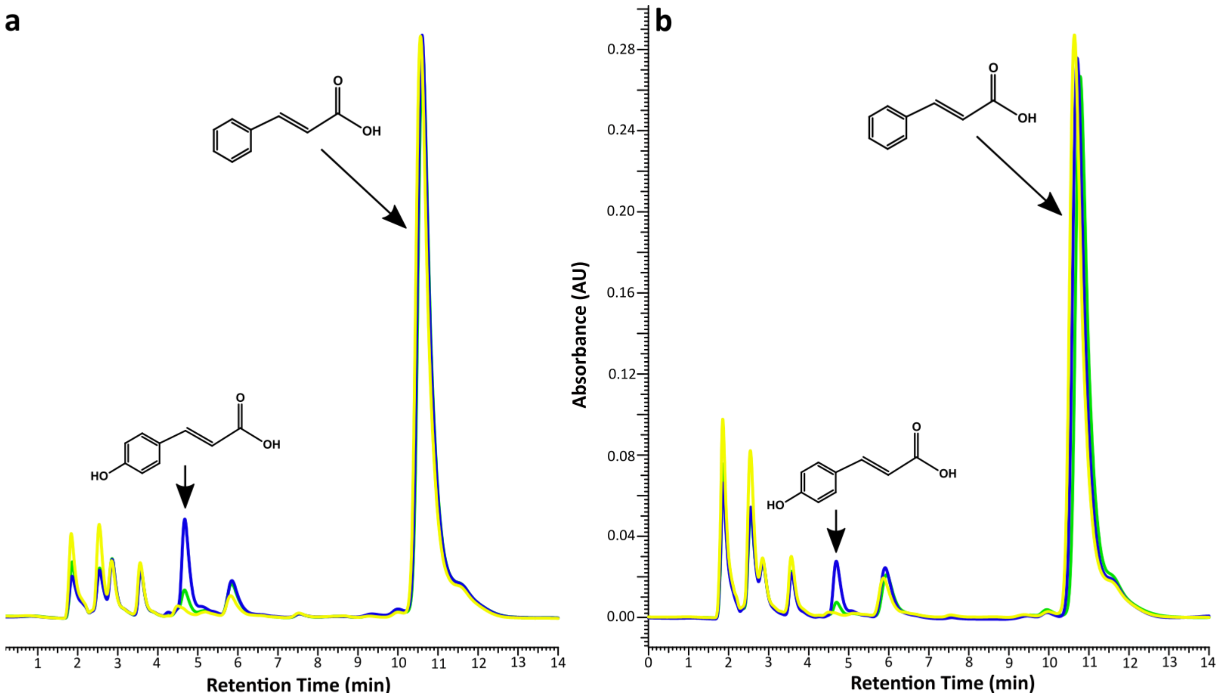

Suppl. Fig. S4 Cosubstrate specificity of Pp_AaC4H (**a**) and Pp_WT (**b**) analysed by HPLC at 309 nm (isocratic elution with 45 % methanol with 0.01 % H_3PO_4). The chromatogram of assays with NADPH as cosubstrate is marked blue, NADH is green and no cosubstrate is coloured yellow

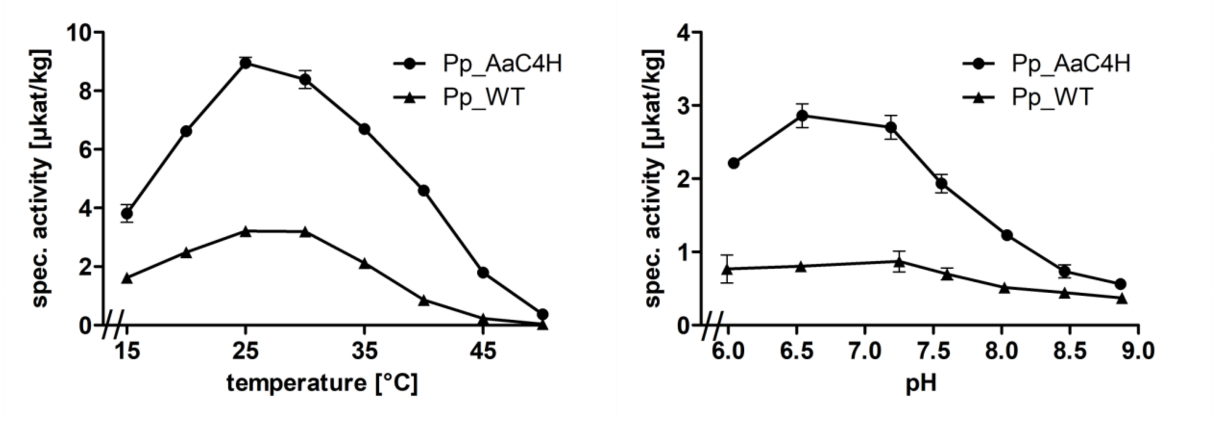

Suppl. Fig. S5 Temperature (**left**) and pH-optimum (**right**) of C4H isolated from Pp_AaC4H and Pp_WT

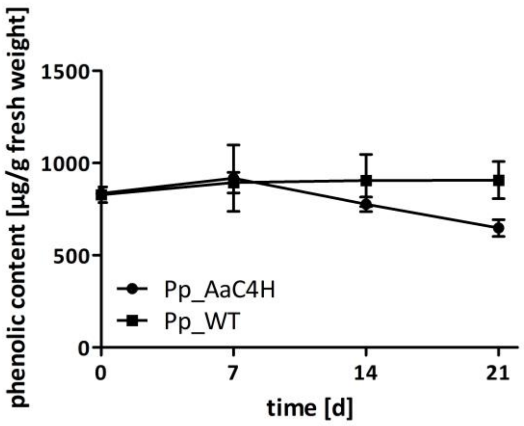

Suppl. Fig. S6 Total phenolic content in ethanolic extracts from suspension-cultured thalli of *Physcomitrella patens* Pp_AaC4H and Pp_WT. Each data point represents the mean average of three samples, the error bars represent SD. A calibration curve of caffeic acid was used for calculation
